# Collectanea Medica, Consisting of Anecdotes, Facts, Extracts, Illustrations, Queries, Suggestions, &c.

**Published:** 1817-03

**Authors:** 


### COLLECTANEA MEDICA,

CONSISTING OF

### ANECDOTES, FACTS, EXTRACTS, ILLUSTRATIONS, QUERIES, SUGGESTIONS, &c.

RELATING TO THE

*History or the Art of Medicine, and the Auxiliary Sciences.*

> Quicquid agunt medici,
> nostri farrago libelli.

### *Biographical Notice of Menuret.*

JEAN JACQUES MENURET, born at Montelimart, in 1733, displayed, from his infancy, the happiest disposition to learning. After having terminated, in a distinguished manner, his course of Humanities, he went to Montpellier to study medicine, which, in that celebrated university, has been almost constantly professed with eclat. Menuret followed, through preference, the lessons of Antoine Fizes, whose extravagant opinions he too often adopted. Adorned with the diploma, he united private study with the public duties of his profession. Diderot and Dalembert, at that period, executed an enterprize sufficient to immortalize them: they laboured, with indefatigable ardour and prodigious talent, in the continuation of the Encyclopædia which they had created. Admitted amongst the number of architects of that grand monument

---

of of French glory, Menuret shewed himself worthy of the glorious choice. The articles he furnished are generally written with great purity of style, and sometimes with elegance: amongst others, we may name Death aud Somnambulism. Others present, in a pure style, paradoxical ideas and inadmissible theories: these are very prominent in the articles Inflammation and Pulse.

The revolutionary tempest troubled the repose of Menuret. The circumstances (says Dr. Montegre) which compelled him to expatriate himself have an historical character, and deserve to be known. He was Dumourier's physician when the Commissioners of the Convention waited upon that General with the order to return to Paris, to have his conduct enquired into. This order was equivalent to that of taking his head to the scaffold. Dumourier, embarrassed by the situation in which he found himself, and, perhaps, not yet decided what to do, on seeing his physician enter the room, said, " Well, Doctor, have you any remedy against this disorder?" " Yes, General," replied the Doctor, " a little recipe of disobedience."* It is well known how the General made use of the recipe; but he published the anecdote, and Menuret, compromised thereby, was obliged to seek an asylum in a foreign land. He chose for his retreat the city of Hamburgh, of which he has described the air, water, &c.

As he had only quitted his native land with regret, he seized the first favourable occasion of returning without danger. The learned societies of Paris, of which he was a member, congratulated themselves on his return, and several others admitted him into their number. The Physician of the Indigent, and a Member of the Committees of Benevolence in his quarter, he filled these functions, at once so interesting and so painful, from choice as well as duty. Those who were intimately acquainted with him assure us, that, when summoned at the same time to attend the couch of princes aud the bed of poverty, he consecrated to the latter his first visit. At the age of seventy he still enjoyed good health, yet one of his medical friends, finding him one day low spirited, asked him if he was unwell?—No, my friend, thank God, I enjoy a tolerable state of health; but it pains me that old age deprives me of one of my sweetest enjoyments, and I cannot mount up to the fifth story.

This venerable philanthropist terminated, in 1815, at the age of 82, a career rendered equally illustrious by his virtues and his talents. He published many excellent works;—that on Contagion is highly curious, though some may object to several of the hypotheses.

"For contagion to take place," says Menuret, " it is necessary that a molecule of matter should be detached from the sick body,

---

* Professor Corcy, the colleague of Menuret in the army commanded by Dumourier, gives the Doctor's receipt differently—" With 2 grains of disobedience, and as much firmness, I am persuaded, General, you will be relieved from it."

and and received by the sound one: and that it should bear the imprint and germ of the disorder, to re-produce in the latter the same order of essential symptoms. These contagious corpuscules are called Miasma; their tenuity hides them from the sight, and we only know them from the effects.

"The small-pox is one of the sinister children which the sun engendered in the stagnant waters of the Nile. The air at first received this new germ, and lodged it on the inhabitants of these infected countries. Carried, by the same means, to other bodies, the seeds germinated, vegetated, and fructified in the same manner, till it has been successively spread, and, as it were, naturalized, in almost all the regions of the known world."

In his essay on the Means of forming good Physicians, and on the reciprocal Obligations of Medical Men and Society (Paris, 1791), he gives a rapid and animated sketch, and, at the same time, a very exact one, of the various branches of the art of healing, and traces with energy the duties he had scrupulously fulfilled.

"Devoted to the solace of suffering humanity, the medical man ought to bind himself never to hear the cry of sorrow in vain, and never to prevent its reaching him, but to fly the moment he hears this imperious signal. At that moment, neither his liberty, his leisure, nor even his existence, is properly his own. Exclusively devoted to the wants of others, he ought to be entirely obedient to them, without any distinction of time or persons. The sacrifice or the interruption of his pleasures, his repasts, and even the hours of rest, are often necessary. It is a tribute which humanity imperiously commands, and woe to him who neglects her voice—woe to him who despises it when it claims his aid in favour of the poor and obscure citizen. But, if he possesses not sensibility?—if he possesses not sensibility! he is not a physician, or ought not to be one; he is only a vile mercenary, a cruel being, making health only a dangerous trade."

### *Medical Bibliography—Winter*, 1816; abridged from Dr. CHAUMETON.

*(Continued from p. 170.)*

I DO not pretend to justify the too mechanical ideas of the immortal Boerhaave on Secretion; but is it necessary equally to reject the plausible theory of Bordeu. This learned physiologist considers every gland as endowed with a kind of sentiment and taste, which only permits the admission of one kind of fluid into its tubes, and excludes all the rest. He places secretion under the immediate influence of a peculiar nervous power, which excites a titillation in the glandular organs, and produces an erection. Bordeu is accused of not having explained himself with sufficient precision, by the reviser (M. Coutanceau). I should be, I confess, less rigid than the author on this point, though I entirely agree with him that the chemists have thrown no light on the nature of semen. This fluid, so remarkable amongst the other wonders of organization, by the property it possesses of communicating to germs, by simple contact, the first vital impulse; for I do not suppose that any physiologist or physician, however he may have been led away by the seductive charms of modern chemistry, can ever so far forget himself as not to recognise the impenetrable mystery in which the re-production of beings is shrouded, and seek an explanation of it in the bottom of a crucible.

Assimilation is the result, the consequence of nutrition, on which, if we are to believe M. Coutanceau, the chemists ought to be silent, notwithstanding their fancying that they have resolved the principal problems relative to this important function. "They do not fail to insist on the notable quantity of phosphate of lime contained in milk; and in which they are resolved to find, as a final cause, the ossifications of a new-born child. I confess I see nothing in this but a barren conclusion. Phosphate of lime appears to me only to be found in milk, because it exists in all albuminous fluids, the reason of which I am equally a stranger to. Muriat of potash is also found in milk, and even in a still greater quantity. Let them, therefore, tell us what is its use in the animal economy. Far from the presence of a calcareous phosphat, in the first aliment of the mammifera, serving to enlighten me on the mechanism of ossification, were I to attach an importance to it which it does not possess, I should only be the more perplexed to conceive how this function could operate in the fœtus, deprived of such a resource; how it is continued after lactation, whatever species of food may be taken into the stomach; and how it is produced in reptiles and birds, which, in the first stages of their existence, are not fed with milk, like the mammiferæ.

There are, however, several phenomena, absolutely chemical, and cited as such by M. Coutanceau, as

1. The colouring of the skin and hair, by being exposed continually to the rays of the sun.

2. The coagulation of albuminous matter, which exudes from a wound or ulcer; and that of the blood itself, which becomes clotted at the extremity of the small vessels, divided by a recent wound.*

3. The existence of soda, in the state of carbonate, in the mucus, while it presents itself, in the form of caustic soda, in all the other albuminous fluids wherein it is met with.

4. The formation of concretions, of various kinds, which are observed in different parts of the animal economy, and particularly in that of the urinary calculi.

5. The action of several poisonous substances, whether on the internal membranes of the stomach or upon one another.

6. The alteration, the acidification, the coagulation, of milk; and the oxidation of metals introduced into the alimentary tube.

---

* We regret much to read this passage from so ingenious an author. What is the chemical process to induce coagulation in this instance?—Edit.

These, it will be seen, are but weak concessions, and even these M. Coutanceau makes with regret, and endeavours to lessen the confidence they might seem to inspire for chemical applications, which are confined, in his opinion, to *surfaces*. "I call exterior, in relation to the animal economy, the phenomena which actually pass in its bosom; and yet nothing can be more exact than this description, for the fluids contained in the grand cavities of the human frame to receive the substances destined for absorption or excretions, as the stomach, the bladder, the intestinal canal, &c. and particularly the bronchial cavities, are evidently susceptible of being brought into immediate contact with the atmosphere, and to communicate with it without the aid of any continued solution. Suppose the natural orifices sufficiently dilated for the external air to penetrate all their depths with the same facility as it touches the exterior of the skin. The fluids contained in these grand reservoirs may be thus considered as external, or out of the body.

Is chemistry, then, capable of serving as a base for the general classification of disorders? M. Coutanceau strongly pronounces in the negative. All the resources of the mind are employed by M. Coutanceau to reduce to their just value, or rather to nothing, the influence of chemistry, which he places far below general physics. "She must not," says he, "servant as she is, and ought to be, set herself up as a mistress, and seek to initiate herself in the mysteries of organization, which are the exclusive patrimony of the physiologist and the physician, &c."

### HEALTH.

It would be improper to state, in a medical journal, that medicine cures but few disorders;—I will therefore simply observe here, that the art of preventing human infirmities appears to me infinitely superior to that of curing them. What homage is not due to the physician who knows how to preserve an unalterable state of health, and prolong and sow flowers in the path of life. I one day propose to publish a work on this subject. I may observe here, that, whatever serves to deteriorate health, ought, as a matter of public morals, to be strictly watched over. I have seen cisterns, wells, bridges, and canals, in such dangerous states and situations, that they actually seemed so many snares laid to produce accidents.

The Romans punished with severity the driver who caused accidents, either by his unskilfulness, or his driving too rapidly. The same law exists in all great capitals. The misfortune is, it is too negligently executed.

The art of poisoning is much better known, and carried to greater perfection, amongst civilized nations, than amongst the savage tribes. They sometimes poison their arrows, with which they combat their enemies; but they have not arrived at the refined villainy of offering, with a calm and even affectionate air, the cup of death to their nearest relatives.

*(To be continued.)*

*Extract from a Paper, by* Sir William Adams, *on the Restoration of Vision, when injured or destroyed, in consequence of the Cornea having assumed a Conical Form.*

[The above having been incorrectly printed in the Journal of Science and the Arts, is now offered in its present form.]

Among the causes producing short sight, is a morbid thickening of the transparent cornea, which has been usually termed Conical Cornea. One of the first, and, I may add, the best descriptions yet given of it, has been by Doctor Léveillé, an eminent French physician, and translator of Professor Scarpa's work on Diseases of the Eye, into the French language.

The conical cornea, although a disease not so frequently met with as many other morbid affections of the eye, is yet by no means of rare occurrence; and any curative effort which is capable of being successfully exerted, becomes the more interesting, the advanced stage of the disease (as far as I have been able to learn) having been hitherto considered by authors as incurable.

It is, therefore, highly gratifying, that the resources of art are found competent to afford effectual relief even in this apparently hopeless case, in which, although it is impossible to remove the disease itself, when thus fully formed, yet, by taking away one of the healthy parts of the eye, whose use is similar to that of the diseased growth of the cornea (which does not admit of removal), vision, it will be shewn, may be restored nearly to perfection.

The malady in question commences by a morbid growth of the whole substance of the cornea, but more particularly its central part, situated opposite to the pupil, and the patient's degree of short sight increases in exact proportion to this growth, which takes place without inflammation, and, in general, without opacity. Its advance, unless arrested by the appropriate medical treatment, is usually slow, but progressive, until, at length, the cornea, instead of being a regular segment of a sphere, wholly losing its natural curvature, assumes a conical form.

This change of structure produces some curious phenomena in the appearances as well as the uses of the cornea. On examining it in front, it assumes an unusual degree of sparkling brilliancy, nearly resembling crystal, except (as is sometimes the case) where there is an opacity in the apex of the cone. Doctor Léveillé attributes this brilliancy to the cornea's strongly reflecting, instead of transmitting, the rays of light; by which he supposes, that the pupil becoming contracted, in a strong light, thereby produces an imperfect and confused sight: but this explanation of the cause of the patient's imperfect vision, it will, however, be hereafter shewn is erroneous. If the cornea be examined laterally, the thickening, it will be observed, gradually increases from its circumference to its centre, where the apex of the cone is usually seated, although, in some instances, I have seen it on one side of the centre. When the cornea is similarly examined, opposite to a strong light, its

 thickness thickness at the base may, in general, be traced, while the sugar-loaf form of the apex renders it impossible to be mistaken for any other disease of the eye.

The change which this disease produces, in respect to vision, is very important. Soon after the commencement of the diseased growth, the patient complains of being unable to see objects distinctly, at the usual distance; and his power of vision becomes gradually shortened, in proportion as the disease advances, until, at length, he is unable to perceive minute objects with any degree of distinctness, however near they may be placed to the eye; and cannot make out large ones when above three or four feet distant. In fact, vision is destroyed in relation to the useful purposes of life, and he becomes nearly as dependant as if totally blind. Indeed, I once saw a young lady labouring under this disease in both eyes, who did not venture to go any where without a guide.

The disease generally begins at the first in one eye, and a similar affection commonly succeeds in the other. I have met with it in almost every stage of life, from a girl of sixteen to an old lady of seventy, and am not aware that it is peculiar to any sex or age, although I have certainly seen it much more frequently in women than in men, and more in young than in old persons.

The opinions generally entertained of the cause of the disease in question, appear to me to have been incorrect, and have necessarily led to an erroneous practice, in the attempts which have been made for its alleviation. The conical form of the cornea has been attributed to an over-distension of that tunic, occasioned by a superabundant secretion of the aqueous humour, which, continually stretching the cornea, has gradually occasioned it to yield to the pressure from within, and thus produced the alteration in its form. To remove this supposed over-distension, it has been usually recommended to evacuate the aqueous humour, by puncturing the cornea, and afterwards to employ pressure—astringent collyria, &c. to prevent its re-accumulation. Experience has, however, shewn the total inutility of these modes of practice. The operation of evacuating the aqueous humour has been, in some instances, repeated several times, without any permanent advantage being found to result from it; and, although it is an operation neither painful nor difficult to perform, yet is sometimes dangerous; for if the crystalline lens should be wounded by the instrument with which the puncture is made, cataract will most likely ensue; and I have been informed of a case, where this actually occurred, during the attempt to evacuate the aqueous humour.

Having, at an early period of my practice, been impressed with the opinion, that the conical form assumed by the cornea in this disease, was the effect of a *morbid growth* of that tunic; and that the short sight experienced by the patient was to be attributed to its increased refractive power, and which, together with that of the crystalline lens, brought the rays of light to a point far short of the retina, it occurred to me, that, as it was impossible to remove the the morbid growth of the cornea, without rendering it unfit for the transmission of light, a useful degree of vision might be restored by the removal of the crystalline lens. I was the more strongly led to form this conclusion, after having myself tried the experiment of looking through deep convex spectacles, such as are employed by patients after the removal of cataract, which, I found, produced a confusion of sight, very similar to that which I had heard described by persons in whom the cornea had been conical in the extreme degree. I, therefore, resolved, more than six years since, while surgeon to the West of England Eye Infirmary, instituted at Exeter, to remove the crystalline lens in a case in which that body had become opake, and also affected with conical cornea. Some circumstances, however, prevented the patient, who was a young woman, and a pauper, from being sent to me to the Infirmary. About three years since, another patient, from the country, an old woman, nearly seventy years of age, placed herself under my care, labouring under this disease, accompanied with cataracts, in whom I successfully removed both the cataracts, and had the gratification to find her vision thereby restored to an extent which far surpassed my most sanguine expectations. I observed that she was capable of seeing much more distinctly without convex glasses than is usual for persons who have undergone the operation for cataract, while, with a convex glass, she could read small print without any difficulty. Not being able to ascertain the degree of vision which this patient experienced previously to the removal of the cataracts, nor whether the diseased change was going on in the cornea and crystalline lens at the same time, I necessarily cannot state the exact amount of the benefit which she derived from the operation. This, however, was demonstrated—that, by the removal of the crystalline lens in eyes affected with conical cornea, nearly perfect vision was restored; while, it is well known, that in cases of conical cornea, where no cataracts exist, vision is usually as imperfect as if the latter malady formed a part of the patient's disease.

The favourable result of this operation fully confirming the opinion which induced me to perform it, I determined, at the earliest opportunity, to try the effect of removing an healthy crystalline lens, as a remedy for blindness produced by conical cornea. A favourable case presented itself the following year, in a young woman, who, during six years, found her sight gradually decreasing, and, at the expiration of that period, had become so blind, from this disease, as to be unable to continue her employment as a servant, and was, in consequence, obliged to apply for parochial maintenance. Shortly afterwards she was sent to an Eye Infirmary in London, where receiving no benefit, she was subsequently brought to me, and solicited, in the most urgent terms, the trial of any practice which afforded a prospect of restoring her to sight. I carefully examined her eyes, and found that the cornea of both eyes had assumed the conical form in a great degree, attended by a slight a slight opacity in the apex of each cone, but none whatever in the crystalline lens. She could walk without a guide, and could see at three or four feet distance, so as to avoid running against any person, but had entirely lost the power of reading, or perceiving minute objects, however nearly they were placed to the eyes.

I effected the removal of the crystalline lens, by causing it to be absorbed, which method of operating is to be preferred to every other hitherto practised, whether the lens be opake or not, in cases where, like the present, it admits of being freely divided. The patient, however, returned to the country before the eye had entirely recovered from the operation; and I did not see her again until nearly twelve months afterwards, when I was in the highest degree gratified to find her capable of discovering minute objects, and reading the smallest sized print, without the assistance of a glass, while holding the book at the usual distance of ten or twelve inches from the eye, nearly as well as she ever recollects to have done. The usual cataract spectacles for near objects, of two inches and a half focus, confused her sight nearly in the same manner as before the crystalline lens was removed, while with those of nine or ten inch foci, her capability of seeing minute objects was somewhat improved. She saw objects at a distance better without than with any glass I could find; whereas the usual standard for distant vision, after the operation for cataract, is four inches focus. She now neither uses a glass for near nor distant objects, has again returned to service; and a gentlemen told me, who has recently seen her, that she accurately described to him an object which was considerably more than a quarter of a mile distant. Twelve months after undergoing this operation, I operated upon the other eye; but she again left town before the eye had recovered itself, and before the lens was entirely absorbed. Previously, however, to her departure, she could read small print with this eye, by the assistance of a convex glass of two and three quarters inches focus, while with one of nine inches focus the sight was greatly improved in viewing distant objects.

In returning to the consideration of the case detailed of the young woman with the conical cornea, it may, perhaps, be supposed, that by admitting the susceptibility of the retina to have been encreased by its being twelve months exercised after the operation, and the adjusting powers of the eye to have been acquired from the same cause, that I abandon my opinion, that the morbid degree of refraction of the light in its passage through the thickened cornea, together with the natural refraction produced by the crystalline lens, were the cause of the confused and imperfect vision previously experienced by the patient; this, however, is not the case, as the fact of the girl being capable of seeing after the removal of the lens, which was not in the slightest degree opake, after having been blind previously, shews clearly that the refractive powers (the conical cornea and crystalline) were too powerful, and that the cure was effected by the removal of one of them. But what I conceive proves the accuracy of of these inductions is, that in the earlier stages of the disease, when the thickening has not attained the height it had reached in the case alluded to, the greatest assistance is afforded to the patient's vision by the employment of concave glasses. It is not, however, my intention to urge, that the refractive power is equally great in the thickened cornea as in the crystalline lens; on the contrary, I think that a convex glass of a less magnifying power than that usually required after the removal of cataract, may be frequently employed to great advantage in cases of conical cornea. Were any further arguments necessary than those adduced to prove that the short sight of the patient is occasioned by the morbid thickness of the cornea, and not by the superabundant quantity of the aqueous humour, as has been supposed, it would be, the well-known fact that water possesses little comparative refractive power, while, from the dense structure and the form of the conical cornea, it is as evident that its powers of refraction must be very considerably increased. Indeed, gutta serena would certainly be produced by the backward pressure of a super abundant quantity of aqueous humour against the vitreous humour and retina, long before it would occasion a protusion of so dense and firm a tunic as the transparent cornea; and I have actually seen gutta serena result from this cause without any material convexity of the cornea being perceptible, although, from over distention, it felt to the touch nearly as hard as an egg-shell.

Although I may have failed in convincing my readers of the accuracy of some of the opinions which I have ventured to submit in this paper, yet I have the gratification of knowing that I have fully proved the important fact, of having successfully carried into effect a mode of treatment capable of restoring vision in a case incurable by other means, and which, as far as I have been able to ascertain, has hitherto never been employed by any other person.

---

*The Vitreous Humour of the Eye capable of Reproduction.*—M. Dreux, of the French Imperial Guard, received in a duel a sabre wound which perforated the right eye, the result was a transverse wound of the cornea with the loss of substance of a small portion of this membrane. This wound, according to Mr. Dreux's account, was instantly followed by the evacuation of a thick limpid liquor, and the flattening of the eye; he was deprived of sight, and felt violent pains, accompanied, at first, by vomiting. He abandoned all idea of ever recovering the sight of his eye; but, to my great surprise, (says Baron Larrey,) the globe gradually resumed its primitive form and natural size; so that it cannot be doubted, that the vitreous humour, of which a certain portion had really exuded, was regenerated; and this fact proves, that Nature can regenerate it wholly, or, at least, in part. The edges of the wound have approximated, and formed an adhesion so light, that the cicatrix is buried without becoming opaque. The wounded iris has resumed its motion, but the pupil remains wounded opposite the cicatrix.

M. Dreux,

M. Dreux, before he left the hospital, saw the light, and, a few months afterwards, he could distinguish colours and objects with the wounded eye.

---

*Dr. Löbenstein Löbel's Proposal of a new Method of Operating for the Cataract.* Communicated by Dr. von Embden.

In operations for the cataract, keratonyxis,* as well as extraction and depression, which the author has had frequent opportunities of performing, the idea struck him, whether extraction might not be performed in that place where ocular surgery directs the depression to be made, viz. one or two lines distant from the cornea through the sclerotica. In the belief of this general notion, he was confirmed by the following most remarkable accident, which happened to a boy about five years of age, at Holycross, a small distanee from Naumburgh. This boy, playing with another, with a penknife, which the other endeavoured to snatch from him, drew it quickly back, and happened to run it into his eye. He set a screaming most violently, and the parents, running to him, immediately sent for the author from Naumburgh. On his arrival, he examined the boy, and found a penetrating incision, of about one-sixth of an inch in length, in the sclerotica, one or two lines from the cornea, towards the bottom of the bulb. The boy having all the time held a folded handkerchief before the eye, the crystalline lens was found to have come out upon it. Between the lips of the wound Dr. Löbel thought he saw small fragments of the lacerated capsule; the aqueous humour had escaped, and the cornea appeared collapsed. Having thus minutely examined the eye, the author considered it as lost, as the sclerotic and choroid coats, the zonula Zinii, and the vitreous humour, must unavoidably have been injured by this penetrating incision. The boy moaning greatly, and complaining of much pain, and being of a plethoric habit, the eye was directed not to be opened; four ounces of goulard-water were prescribed, in which cloths were to be dipped, and applied lukewarm to the closed eye. An emulsion of poppies, with nitre, was also prescribed, an antiphlogistic regimen inculcated, and all solid food prohibited for eight days. He was also ordered to lie rather low, on his back or left side; and the only window in the room in which he lay was obscured by cloths, besides the shutters being shut, so that even the smallest ray of light was excluded. Sugar water, with some cream of tartar, was the only beverage he was permitted to take.

On visiting the boy again, on the third day after the accident, Dr. L. was greatly surprised to find, that he felt not the least pain in the eye, and that his pulse was not at all feverish, but quite regular.

* Puncturing of the cornea would, to an English ear, be more musical than the above Greek combination, so full of German gutterals.—Edit.

regular. The eye-water was ordered to be continued but twice a-day, the emulsion with the nitre was omitted, but the cream-of-tartar drink persevered in. After four days more, the boy was allowed to get out of the bed, and sit sometimes in a chair; but he was desired to be kept from stooping, or leaving the dark chamber. The diet prescribed was to be continued until the tenth day, nor did Dr. L. venture to open the eye before then. On re-visiting the patient on the tenth day from the accident, a little day-light being let in through the window into the patient's room, he placed the boy with his back to the window, and then cautiously and fearfully opened the injured eye, covering the healthy one with his right hand, and asking the child, Whether he could see? How agreeably was he surprised to find that he could not only distinguish the white wall, but also a whip hanging on the same. He now permitted him to see for about four or five minutes in the same position, in order that the affected organ might not be too violently affected by the operation of the light, but become gradually accustomed to it; then, turning the boy more with his left side towards the window, he examined the condition of the morbid eye, and found that the cornea had almost recovered its convexity, but that the pupil was somewhat larger than that of the healthy eye; the albuginea was rather red, and only a bluish red streak shewed itself in that place where the penetrating incision had happened. The eye was now tied up again with a dry compress, the fluid regimen continued, and the boy ordered to remove the bandage every morning for half an hour, and to see with the eye under a green eye-screen. On visiting him again after six days, the cornea had fully recovered its natural shape, the pupil was of the same size with the other, the bluish red streak had gone off, and the patient, seeing clearly and distinctly all objects, was discharged as perfectly cured, the author being much astonished at his not having lost his eye and vision.

This fact, however, corroborated the author in the idea, that depression and extraction might be made much easier through the conjunctiva than through the cornea. He thinks this operation promises the following advantages:

1. It would be chiefly indicated in cases where neither depression or keratonyxis can be made with success, *e. g.* when the capsule, half or a whole line thick, is morbidly indurated, or when the lens is very large, and cannot be broken down.

2. Neither prolapsus of the iris nor iritis would be to be dreaded, which, in extracting the cataract through the cornea, is frequently the case.

3. The re-union of the wound would be more expeditious than on making the incision through the cornea, as the upper and lower eye-lids would close the lips of the wound more intimately. The incision in the bulb should be made many lines lower down in the conjunctiva than the depressing needle is commonly thrust in, and this method might thus be called extraction through the posterior chamber.

 4. The

4. The point of the knife used in extraction should be pressed and moved towards the lens, and, in order not to injure the iris with it, it should be carried vertically through the eye, right opposite the place where it entered, when, instead of applying the pressure usual in the common method of extraction, in order to carry the lens through the pupil and opened cornea, the upper eye-lid would only want a little rubbing, and a gentle pressure. By this manipulation, the lens will, for a certainty, descend through the incision and the conjunctiva, and the capsule follow it, or at least the latter will be absorbed if it be thin, and lacerated by the point of the knife. Should, however, large fragments of the capsule be perceived to swim about, they might be carefully removed by a small pair of pincers.

Dr. L. thus proposes this method in such cases where keratonyxis and depression are contra-indicated, and where extraction is the only resource, or where adhesions of the crystalline exist. As to the objections which might be taken from the unavoidable lesion of the conjunctiva, sclerotica, zonula Zinnii, and corpus vitreum, he is persuaded from experience, that, by proper management, and due medical care, these cannot be so dangerous as to influence us; for, in the first place, if they were so, the greater number of depressions must prove much more unsuccessful, which is really the case; and, secondly, in the above case of injury with the penknife, in which he most likely pressed the crystalline through the wound by the pressure he caused by holding the folded handkerchief before the eye, and where he, by his crying and screaming, caused determination to the head, and the injured organ, the boy must have lost his eye and vision, through the combination of such untoward circumstances.

The author concludes with conjecturing, that Kerkringius, Burhius, Taylor, and Woolhouse, made use of this method, when they boasted of having restored a young and acute vision to aged people, by removing the corrupted and turbid humours of the eye, and replacing new ones in their stead.

---

We some time ago promised a few more extracts from Mr. Bedingfield's valuable selections. The last of the following cases will be found particularly interesting.

"A young woman, eighteen years of age, was suddenly attacked with the symptoms of acute peritonitis. She complained of extreme pain over the whole abdomen, which was greatly aggravated by pressure. Her pulse was hard, small, and frequent; her countenance anxious; her tongue white; her skin hot and dry. From thirty to forty ounces of blood were taken away for five days in succession, and afterwards a few ounces occasionally, when a febrile disposition was perceptible. The total quantity of blood abstracted was two hundred and forty ounces.—She remained in a debilitated state for some weeks, but ultimately perfectly recovered.

"A boy, aged seventeen years, labouring under precisely similar symptoms, had fallen under my observation a short time previously. I never saw two cases bear a more striking resemblance to each other.

"Sixteen ounces of blood were taken from his arm; some leeches applied to the abdomen, and half an ounce of the infusion of digitalis was directed every six hours. The boy died at the end of three days from his first seizure. The digitalis produced no effect either upon the pulse, head, or stomach.

"The peritonæum and intestines exhibited traces of the most active inflammation. The intestines were glued together; flakes of coagulable lymph were deposited upon their surfaces; and nearly a gallon of serum had been poured into the cavity of the abdomen, in which globular transparent pieces of lymph were also floating.

"*Remarks.*—The violence of the pain, tenderness, &c. form the best criteria for estimating the danger of the patient; and our practice must be regulated by their intensity. If we pay any regard to the pulse, we shall be more frequently deceived than assisted by it. I have seen cases of peritonitis, in which it remained perfectly natural; others, in which it was as strong and full as in pneumonia, acute rheumatism, or any active inflammatory disease; others in which it was small, feeble, fluttering, and almost imperceptible.—A remarkable instance of the insidious nature of the disease happened about two years ago.

"A young woman, for five days after a natural labour, appeared to be getting well without the occurrence of one unpleasant symptom.*

"On the evening of the fifth day, she complained of a little tenderness above the pubes, slight nausea, and the abdomen felt rather tense. The pulse was soft and equal; the tongue clean; the skin moist; the bowels confined. She was directed to take an aperient. For three succeeding days she remained in nearly the same state; her pulse then sunk so low as to be scarcely perceptible; the surface of the body became cold, and covered with a clammy perspiration; the abdomen tense and inflated; and the stomach rejected every thing. Cordials were directed for her, but she died in thirty-six hours.

"*Examination.*—The left appendages of the uterus were found in a sphacelated state; sphacelated spots were seen upon various parts of the intestines, the peritonæum was almost universally inflamed, coagulable lymph thrown out, and several quarts of serum were in the cavity of the abdomen. No disease of the uterus

"* After delivery, the uterus did not contract upon the placenta with sufficient force to effect its expulsion; and the funis was so very slender that it would have given way, had efforts for the extraction of the placenta been made with it. I was, therefore, under the necessity of detaching it with my fingers from the uterus."

 existed; existed: it had not, however, returned to its natural dimensions in the unimpregnated state.

"*Observations.*—This unfortunate case ought to teach us to give our utmost heed to any tenderness or pain complained of in the abdomen after delivery, however slight it may be. As a precautionary measure, we shall do well to make use of frequent gentle pressure, to ascertain whether or not there be any tendency towards inflammation. This woman, previously to her confinement, had been much debilitated by excessive discharges from the vagina, and from the ulcerated surface of a large warty excrescence upon the pudenda. This consideration, together with the total absence of inflammatory symptoms, forbade the use of the lancet; or, perhaps, I ought rather to say, deterred her attendants from having recourse to it, or from supposing that any necessity for it existed."

---

### CRITICAL ANALYSIS

### *OF RECENT PUBLICATIONS*

IN THE

